# Frequency of intravascular large B-cell lymphoma in Japan: Study of the Osaka Lymphoma Study Group

**DOI:** 10.1186/1756-8722-4-14

**Published:** 2011-04-11

**Authors:** Takeshi Chihara, Naoki Wada, Junichiro Ikeda, Shigeki Fujita, Yumiko Hori, Hiroyasu Ogawa, Haruo Sugiyama, Shosaku Nomura, Itaru Matsumura, Masayuki Hino, Yuzuru Kanakura, Eiichi Morii, Katsuyuki Aozasa

**Affiliations:** 1 Departments of Pathology, Osaka University Graduate School of Medicine, 2-2 Yamadaoka, Suita, Osaka, Japan; 2 Department of Internal Medicine, Hyogo College of Medicine, 1-1 Mukogawa-cho, Nishinomiya City, Hyogo, Japan; 3 Departments of Functional Diagnostic Science, Osaka University Graduate School of Medicine, 2-2 Yamadaoka, Suita, Osaka, Japan; 4 First Department of Internal Medicine, Kansai Medical University, 10-15 Fumizono-cho, Moriguchi City, Osaka, Japan; 5 Department of Hematology, Kinki University School of Medicine, 377-2 Oonohigashi, Sayama City, Osaka, Japan; 6 Department of Clinical Hematology and Diagnostics, Osaka City University Graduate School of Medicine, 1-5-7 Asahi-cho, Abenoku, Osaka, Japan; 7 Departments of Hematology and Oncology, Osaka University Graduate School of Medicine, 2-2 Yamadaoka, Suita, Osaka, Japan

## 

Dear Editor:

Intravascular large B-cell lymphoma (B-IVL) is listed as a distinct disease entity in the World Health Organization(WHO) classification for lymphoid neoplasms [1]. The disease is rare, and information for its exact frequency among non-Hodgkin lymphoma (NHL) has been extremely limited. There have been several reports mainly from Asian countries describing the different disease frequencies [2-4]. In addition, population of the cases was variously described among these reports. Therefore it is difficult to know the geographical difference in disease frequency, which might be helpful for understanding pathogenesis of disease.

From November 1999 to December 2010, a total of 5,085 cases were registered with the Osaka Lymphoma Study Group (OLSG), which was established in 1999, and more than 60 institutes in Osaka area, Japan participate to it. All of the hematoxylin and eosin-and immunoperoxidase-stained sections were reviewed by one of the authors (KA). A diagnosis of malignant lymphoma was confirmed in 4,066 cases (80.0%). Of these 4,066 cases, 3,726 (91.6%) were NHL and 340 (8.4%) Hodgkin lymphoma. Number of cases with DLBCL and B-IVL was 1,815(44.6% of all ML, 48.7% of NHL) and 9(0.22% of all ML, 0.24% of NHL), respectively. Histologic criteria for the diagnosis of B-IVL is selective growth of B-lymphoid cells, usually with large size, within lumina of small or intermediated vessels.

9 cases of B-IVL in OLSG were summarized in Table 1.The median age of the B-IVL patients (67 years old) at biopsy for diagnosis in the present series was similar to that of the previous reports from Japan and Italy, but slightly older than that from Thailand [4-6]. Females predominated males remarkably in the present series. Frequency of presence of neurological and B-symptoms was rather similar among present and the previous reports from Asian countries and Italy [4-6]. Whereas cutaneous lesions were more frequently found in Italy compared to Asian countries. Lymphadenopathy and hepatosplenomegaly were more frequently found in Asian than in Western cases [4-6]. Recently, Japanese investigators reported that frequencies of neurological symptoms and cutaneous lesions were low but that of hemophagocytic syndrome was high in B-IVL of Asian countries, and advocated the term "Asian variant of B-IVL" [5,7-10]. But as shown in Table 2, these findings were not discriminating ones to distinguish Asian from Italian cases into different category. It is rather a difference of frequency, not justifying B-IVL in Asian countries as Asian variant. The frequency of hemophagocytosis was relatively low (19%) in Sanya et al's cases from Thailand, whereas it was high (61%) in Murase et al's cases [4,5]. The common site for biopsy was lung in the present series, whereas bone marrow in Murase's and Sanya's series [4,5]. The present patients were treated with chemotherapy mostly with rituxan. Two patients received auto peripheral blood stem cell transplantation and radiotherapy. Follow-up duration in 8 patients diagnosed by biopsy ranged from 18.6 to 111.8(median 30.6) months for the 5 survivors. Three-year survival rate was 67%.

**Table 1:** Brief summary of 9 cases of intravascular large B-cell lymphoma

Case	Age	Sex	Site	Treatment	Outcome (survival time)
1	60	f	lung	unknown	alive (111.8 months)
2	79	f	nasal cavity	THP-COP	dead
3	55	f	retroperitoneum	R-CHOP, auto PBSC, Radiation	alive (81.3 months)
4	62	f	lung	R-CHOP, auto PBSC, Radiation	alive (52 months)
5	69	f	lung	R-THP-COP	dead (30.6 months)
6	93	f	liver/spleen	Not done	Autopsy case
7	74	m	kidney	unknown	unknown
8	40	f	skin	R-CHOP	alive (20.0 months)
9	67	f	lung	unknown	alive (18.6 months)

f:female, m: male

THP-COP: pirarubicin, cyclophosphamide, vincristine and prednisolone

R-CHOP: rituximab-cyclophosphamide, doxorubicin, vincristine, and prednisone

R-THP-COP: rituximab-pirarubicin, cyclophosphamide, vincristine and prednisolone

PBSC: peripheral blood stem cell transplantation

**Table 2:** Brief summary of intravscular large B-cell lymphoma

		Number of cases	Age (y)	Sex	Symptoms			Physical findings		
						
Source	Publication Country	B-IVL/total (%)	range (median)	(M/F)	neurological	cuteneous	B-symptoms	lymphadenopathy	hepatomegaly	splenomegaly
Present	Japan	9/3726(0.24%)	40-93(67)	1/8	2/7(29%)	1/7(14%)	8/9(89%)	3/8(38%)	5/8(63%)	7/8(88%)
Murase	Japan	96/nd	41-85(67)	50/46	26/96(27%)	14/96(15%)	73/96(76%)	nd	53/96(55%)	64/96(67%)
Ferreri	Italy	38/nd	34-90(70)	18/20	13/38(34%)	15/38(39%)	21/38(55%)	4/38(11%)	10/38(26%)	10/38(26%)
Sanya	Thailand	17/1286(0.93%)	34-77(59)	7/10	10/17(24%)	2/17(12%)	11/17(65%)	4/17(24%)	12/17(71%)	13/17(76%)

nd: not described

The frequency of B-IVL among NHL was 0.24% in the present series, and that in the previous reports from Japan, Hong Kong, and Thailand was 0.1%, 0.31%, and 0.91%, respectively [2-4]. Whether the differences in frequency among Asian countries might be due to collection bias, i.e., use of randomly collected cases, inclusion of consultation cases, or serially registered cases, or reflect real difference in the disease incidence among these countries could not be judged based on the data shown in the previous literature. Present study is based on the serially registered cases with the OLSG, mostly reflecting the real frequency of B-IVL in Japan.

According to the statistics (Center for Cancer Control and Information Services, National Cancer Center, Japan, 2005), approximately 15,560 persons are newly diagnosed in Japan as having NHL annually [11]. Based on the data presented by the Lymphoma Study Group of Japanese Pathologists [3] or the OLSG, it is postulated that only 16 or 37 persons are diagnosed as B-IVL in Japan annually. When adopting the former data, 16 persons, a whole of newly diagnosed cases in Japan for approximately 6 and 12.5 years must be collected to obtain approximately 100 and 200 cases of B-IVL, respectively. For this, extensive collaborative study is indispensable.

In conclusion, formal epidemiologic study is necessary to know the difference of B-IVL among each area. There are some differences in frequency of symptoms among each district, but it does not necessarily mean the presence of specific variant in some area.
